# Supplementary material for: Pig genome functional annotation enhances the biological interpretation of complex traits and human disease
Source: Nat Commun. 2021 Oct 6;12:5848. doi: 10.1038/s41467-021-26153-7 (PMC8494738; doi:10.1038/s41467-021-26153-7)
Supplement: Supplementary file 1 — Supplementary Information [file 41467_2021_26153_MOESM1_ESM.pdf]

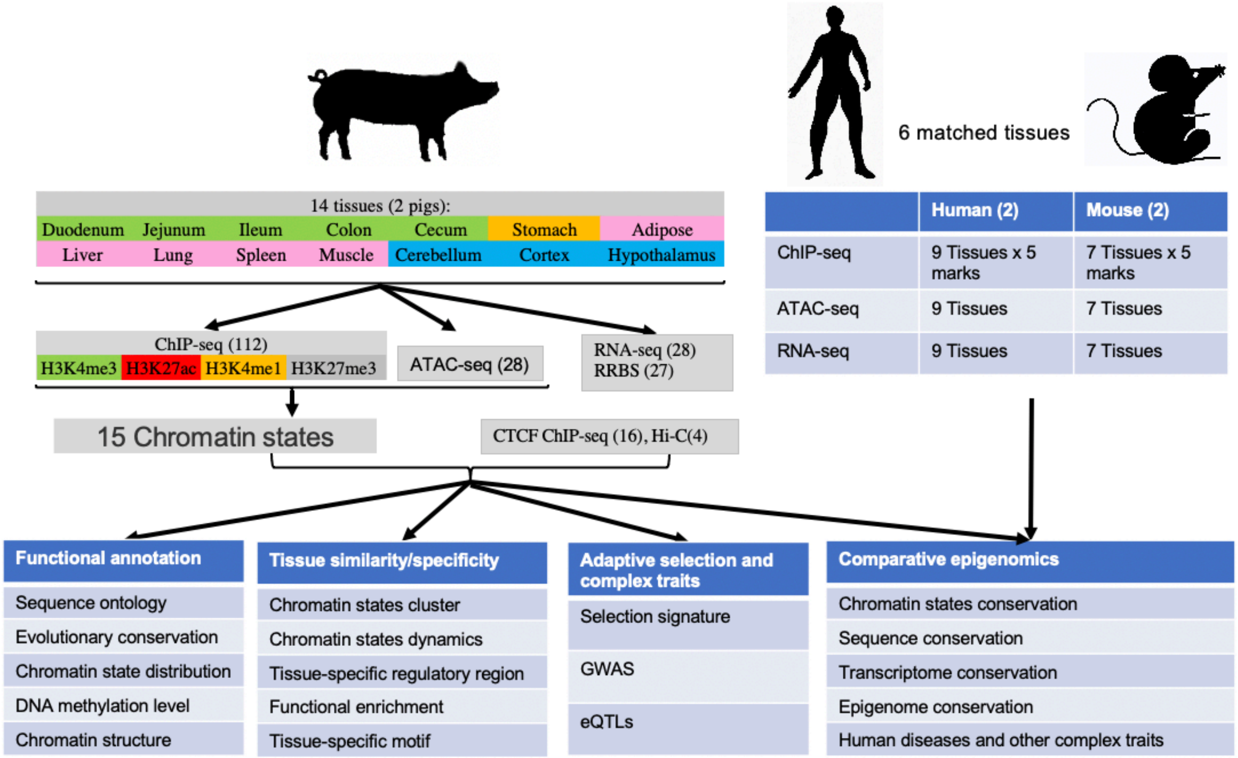

Supplementary Fig. 1 Study design of pig genome annotation and comparative epigenomic analysis. This study included 112 histone ChIP-seq, 28 ATAC-seq, 28 RNA-seq, 27 RRBS, 16 CTCF ChIP-seq, 4 Hi-C datasets cross 14 tissues from pig, and also integrated 126 human and 98 mouse datasets for comparative epigenomic analysis. The six matched tissues among pig, human, and mouse were small intestine, liver, spleen, lung, adipose, and brain cortex. The numbers in the brackets are the number of assays or samples included in the current study. 16 CTCF ChIP-seq of the same two biological replicates of eight core tissues (Adipose, Cerebellum, Cortex, Hypothalamus, Liver, Lung, Muscle, Spleen) from our FAANG pilot project (PRJEB14330)[1], and four liver Hi-C of large white pig from publicly available dataset (PRJEB27364)[2].

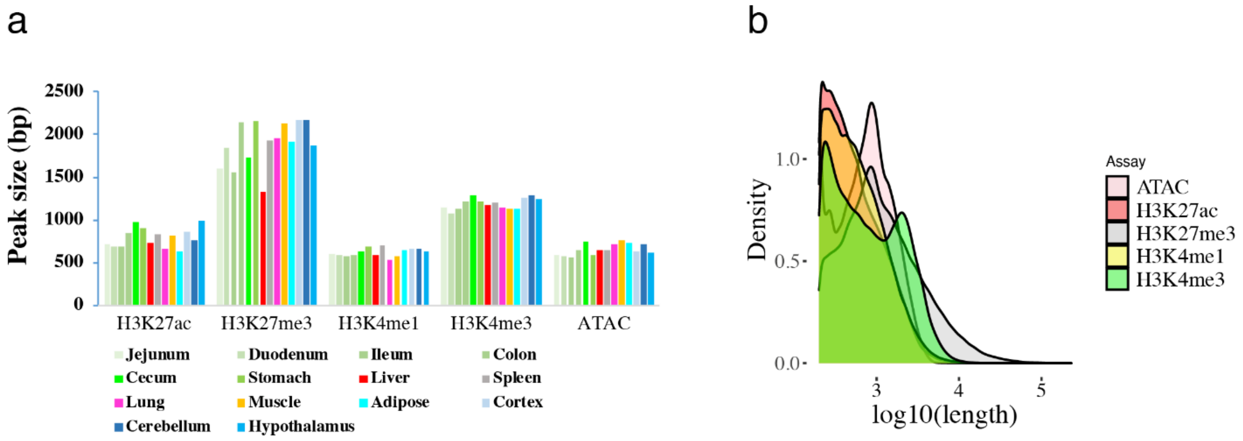

Supplementary Fig. 2 Peak size and size distribution of epigenetic marks. a, Average peak size for each epigenetic mark in each tissue. b, Peak length distribution of epigenetic marks in adipose tissue.

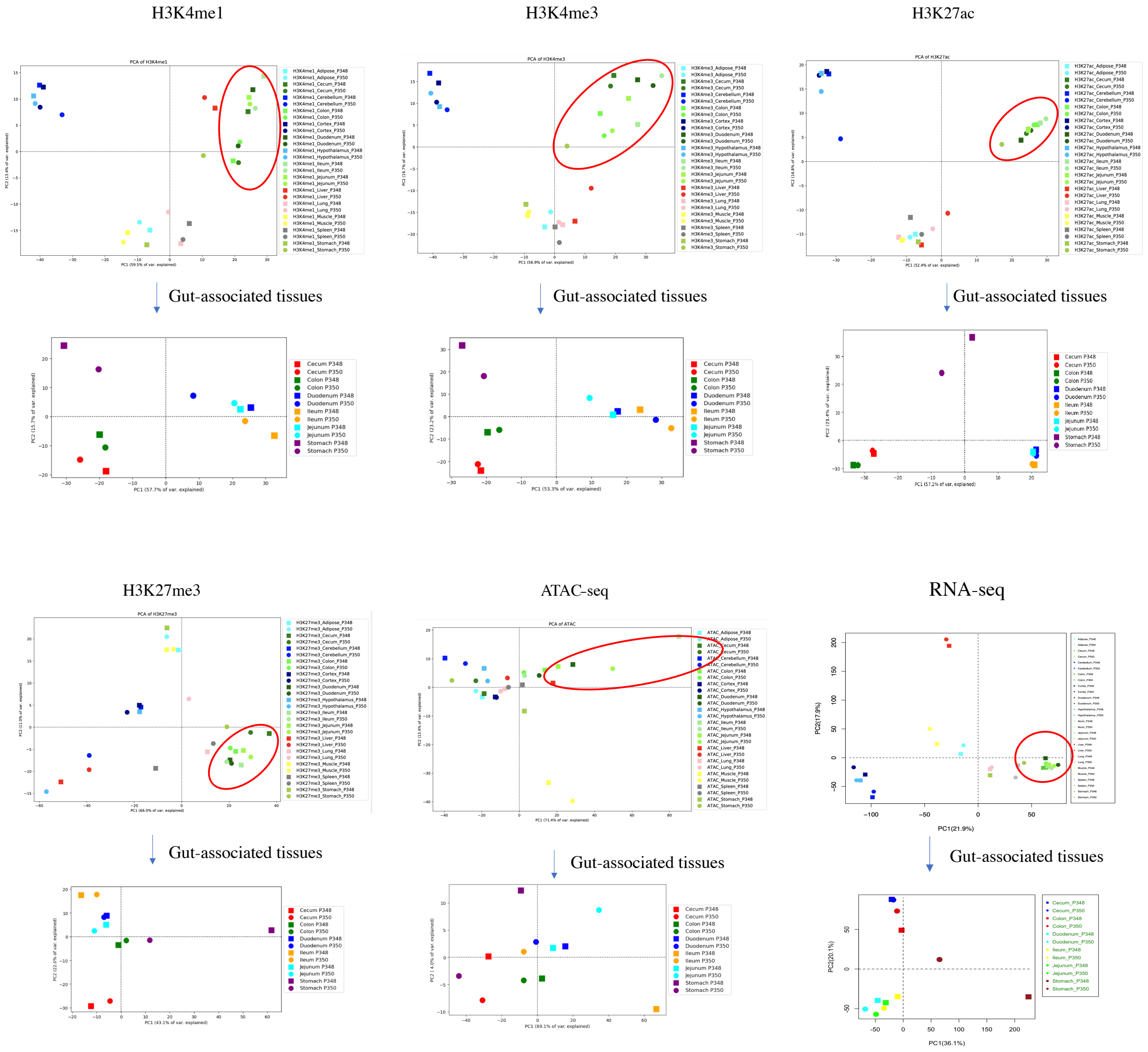

Supplementary Fig. 3 Principal component analysis (PCA) of five epi-marks and RNA-seq in 14 tissues in pig. The regions highlighted are gut-associated tissues.

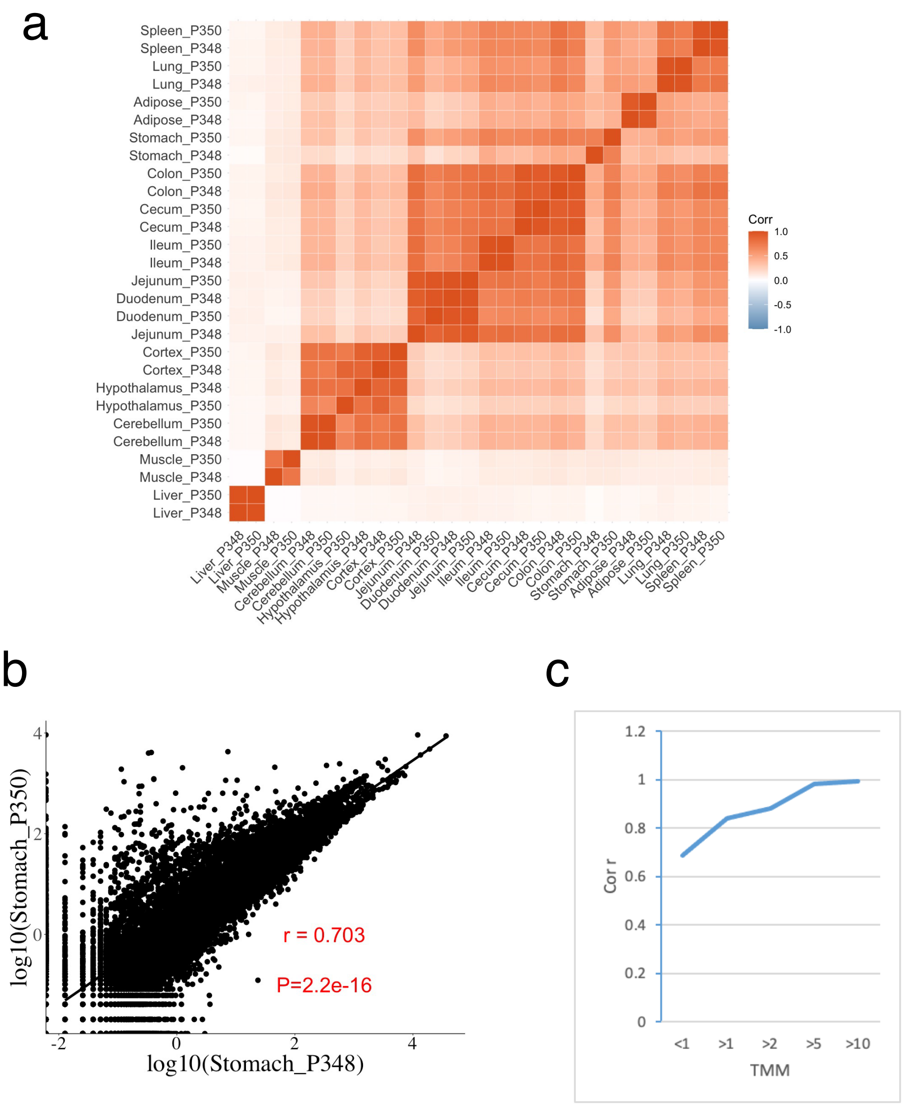

Supplementary Fig. 4 Correlation aomng RNA-seq samples. **a,** Correlation of all RNA-seq samples based on TMM. Hierarchical clustering by hc.method = "complete". **b,** The correlation plot of two RNA-seq data from stomach tissue based on TMM. The significant test base on two-sided *t*-test. **c,** Correlation coefficiency (r) of two stomach RNA-seq data at different expression level of genes. >1 means TMM of alll genes from two replicates of stomach RNA-seq over 1. In contrast to PCA analysis, the hierarchical clustering analysis (hc.method = "complete") of all RNA-seq samples showed the two stomach samples were clustered together. The correlation

result showed the expression levels of all genes between the two stomach samples were significantly correlated (r=0.703; p-value $< 2.2e-16$). In addition, as the gene expression level increases, the correlation between two samples becomes higher. It reaches 0.982 when TMM > 5 (n = 10,269).

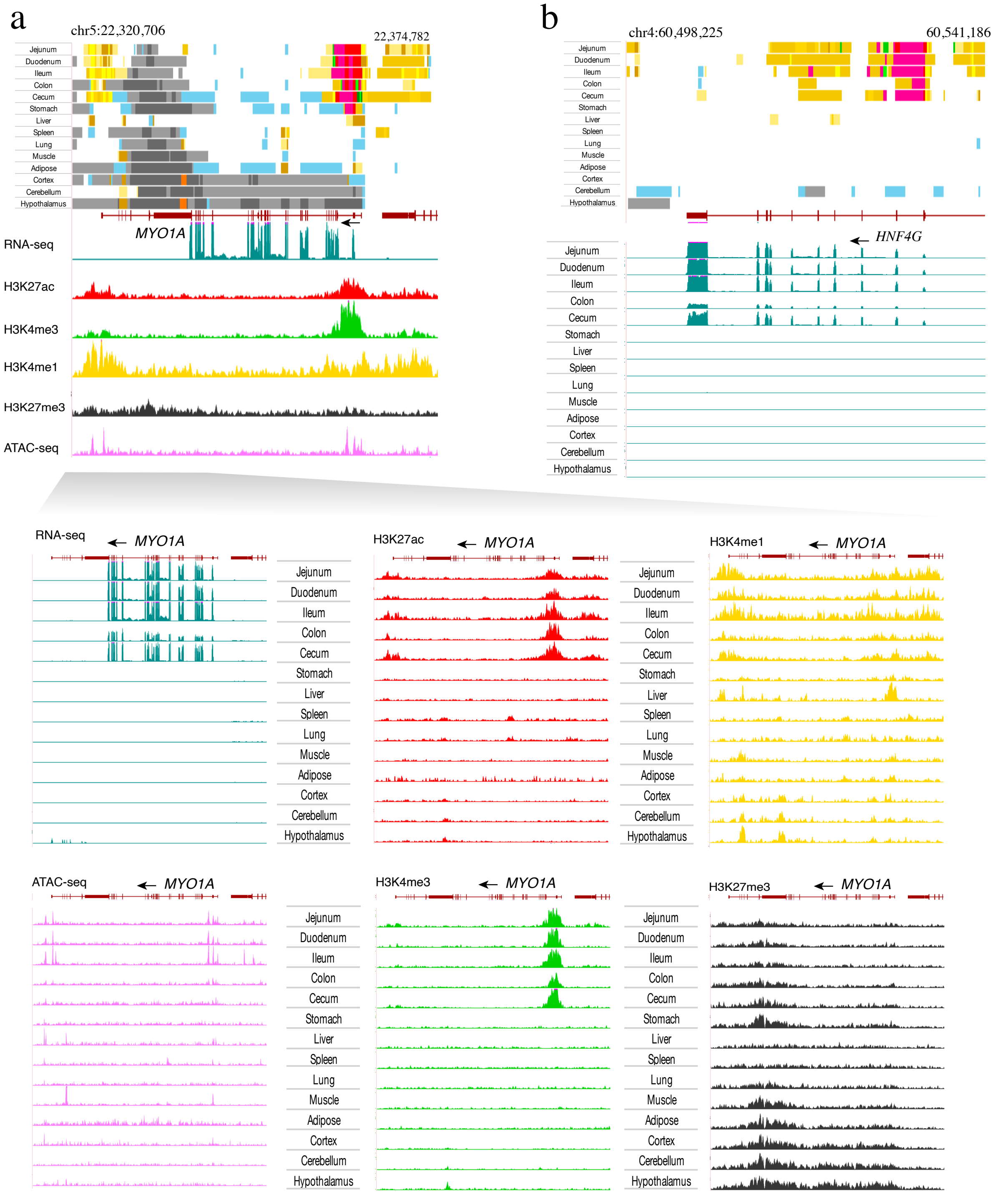

Supplementary Fig. 5 Example of regulation of gene expression by chromatin states. **a**, Chromatin state around *MYO1A* gene (chr5:22,320,706-22,374,782) in 14 tissues. **b**, Chromatin

state around *HNF4G* (chr4:60,498,225-60,541,186) in 14 tissues. Vertical scale 0-200 for RNA-seq, 0-100 for H3K27ac and H3K4me3, and 0-50 for other marks and ATAC-seq.

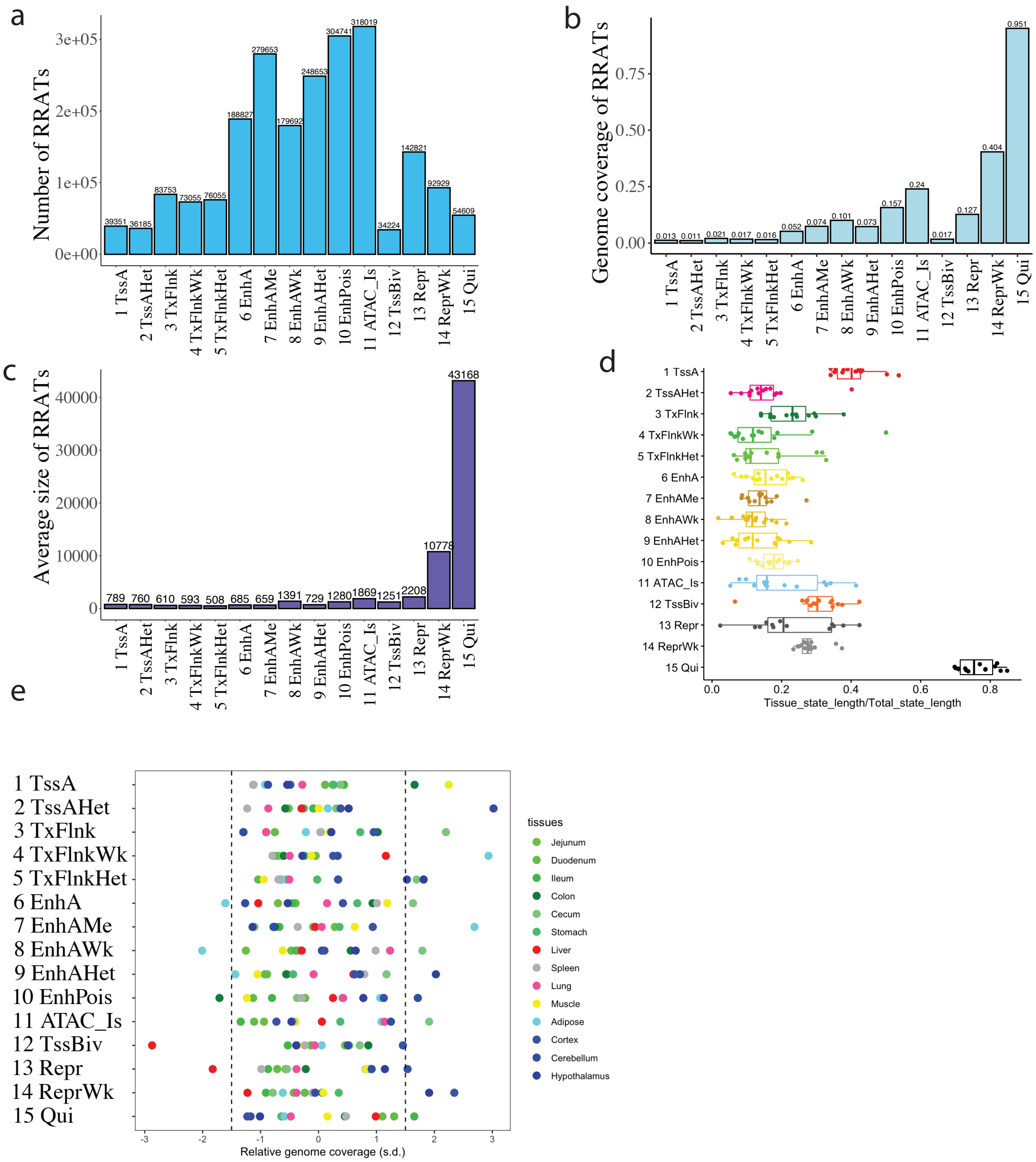

Supplementary Fig.6 **Total number, genome coverage and characterizations of regulatory elements at each chromatin state across 14 tissues. a**, Total number of regulatory regions across 14 tissues (RRATs) for each chromatin state. Totally we identified 2,097,958 regulatory elements (exclude Qui) spanning 14 tissues including 39.351 active promoters (TssA), 188,827 active strong enhancers (EnhA), and 142,821 repressors (Repr). **b**, Genome coverage of RRATs

for each chromatin state. **c**, Average size of RRATs for each chromatin state. **d**, Average genome coverage for each chromatin state in 14 tissues. Whiskers show 1.5× interquartile range. Each circle presents one of 14 different tissues. **e**, Chromatin state relative genome coverage in different tissues.

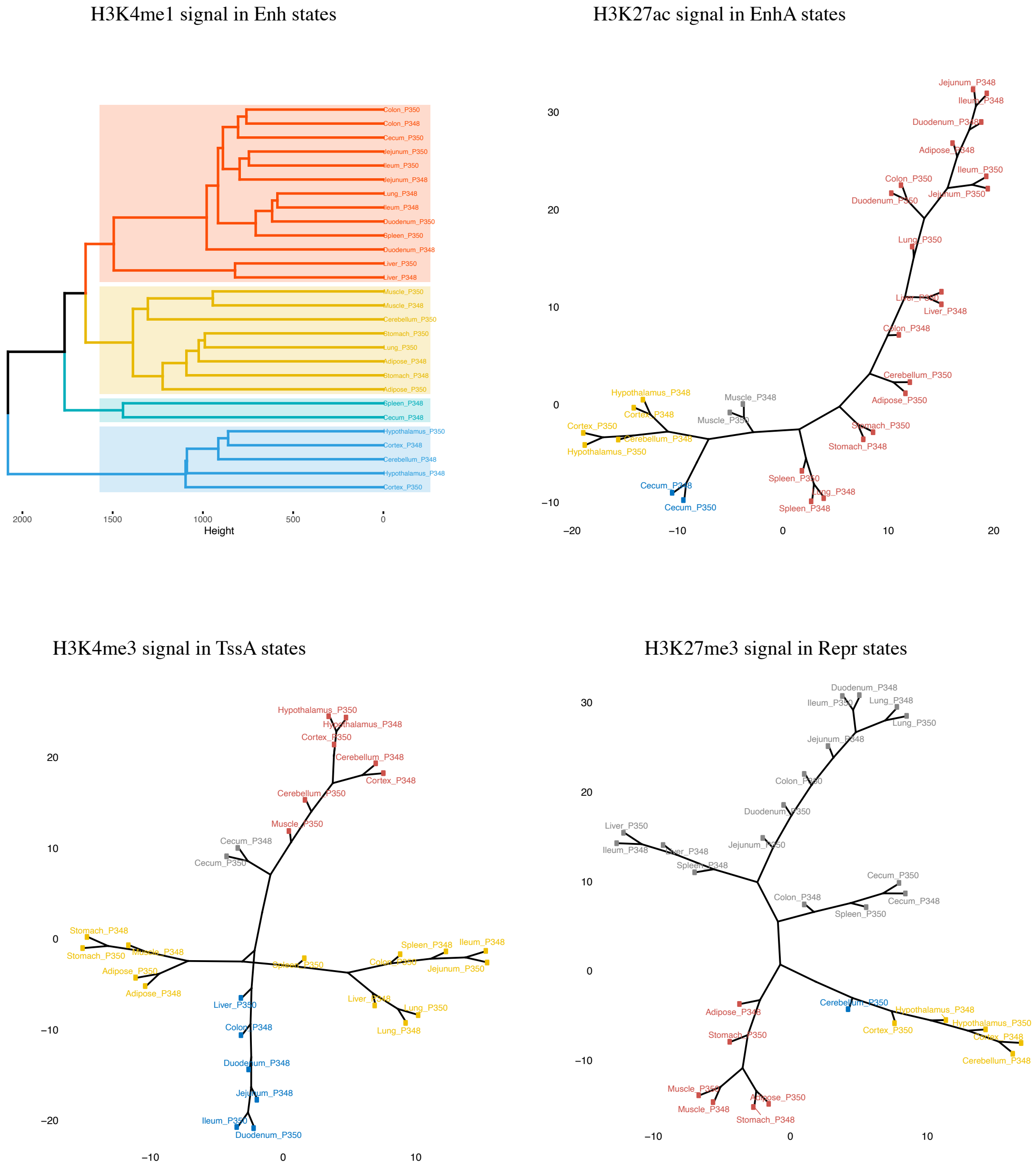

Supplementary Fig.7 Hierarchical epigenome clustering using H3K4me1 signal in EnhA states, H3K4me3 in TssA, H3K27me3 in Repr, and H3K27ac in EnhA

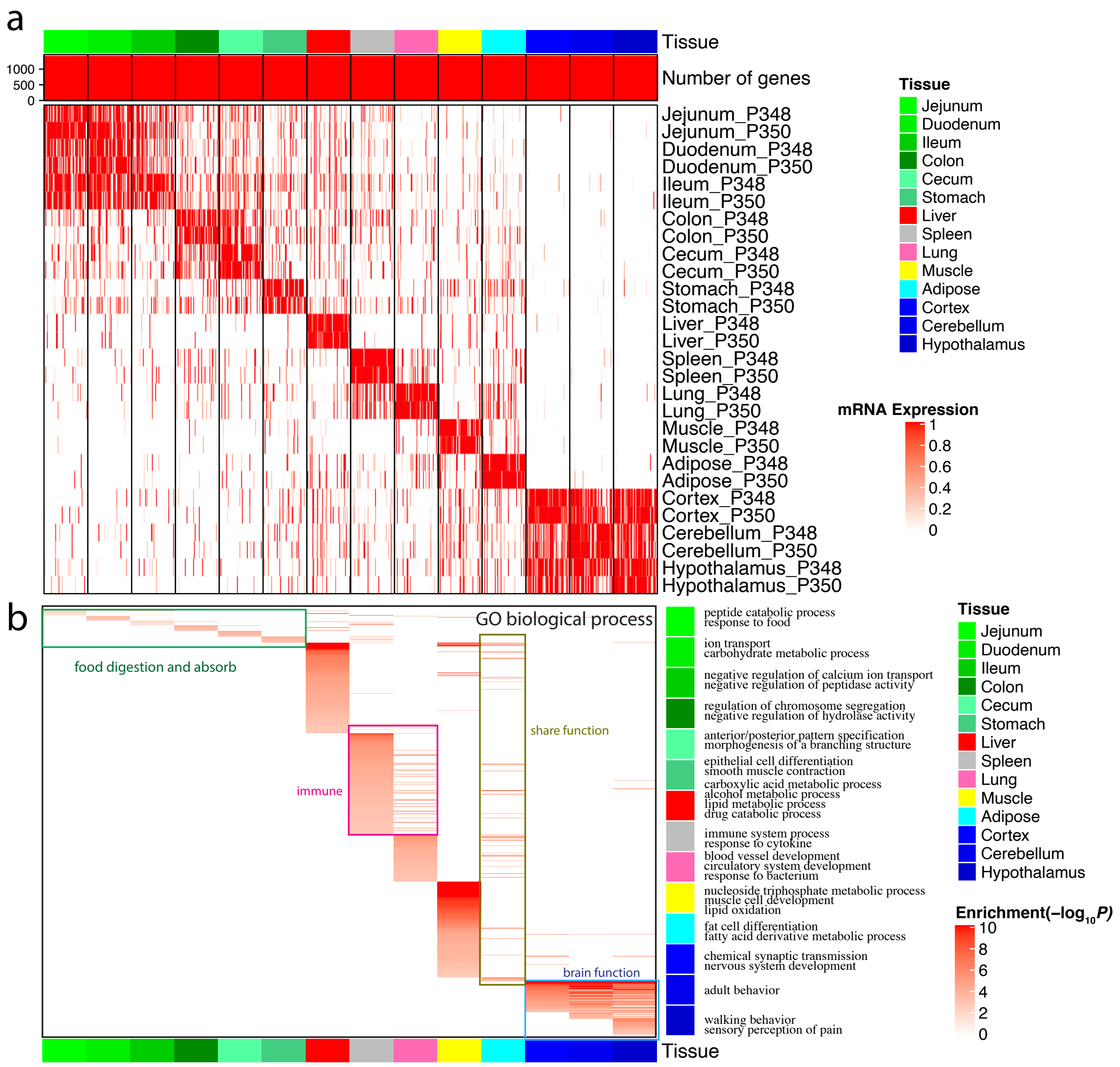

Supplementary Fig. 8 The expression of tissue-specifically expressed genes (TSE) across 14 tissues (**a**) and their gene ontology (GO) functional enrichment (**b**). The *P* value was generated by GREAT program.

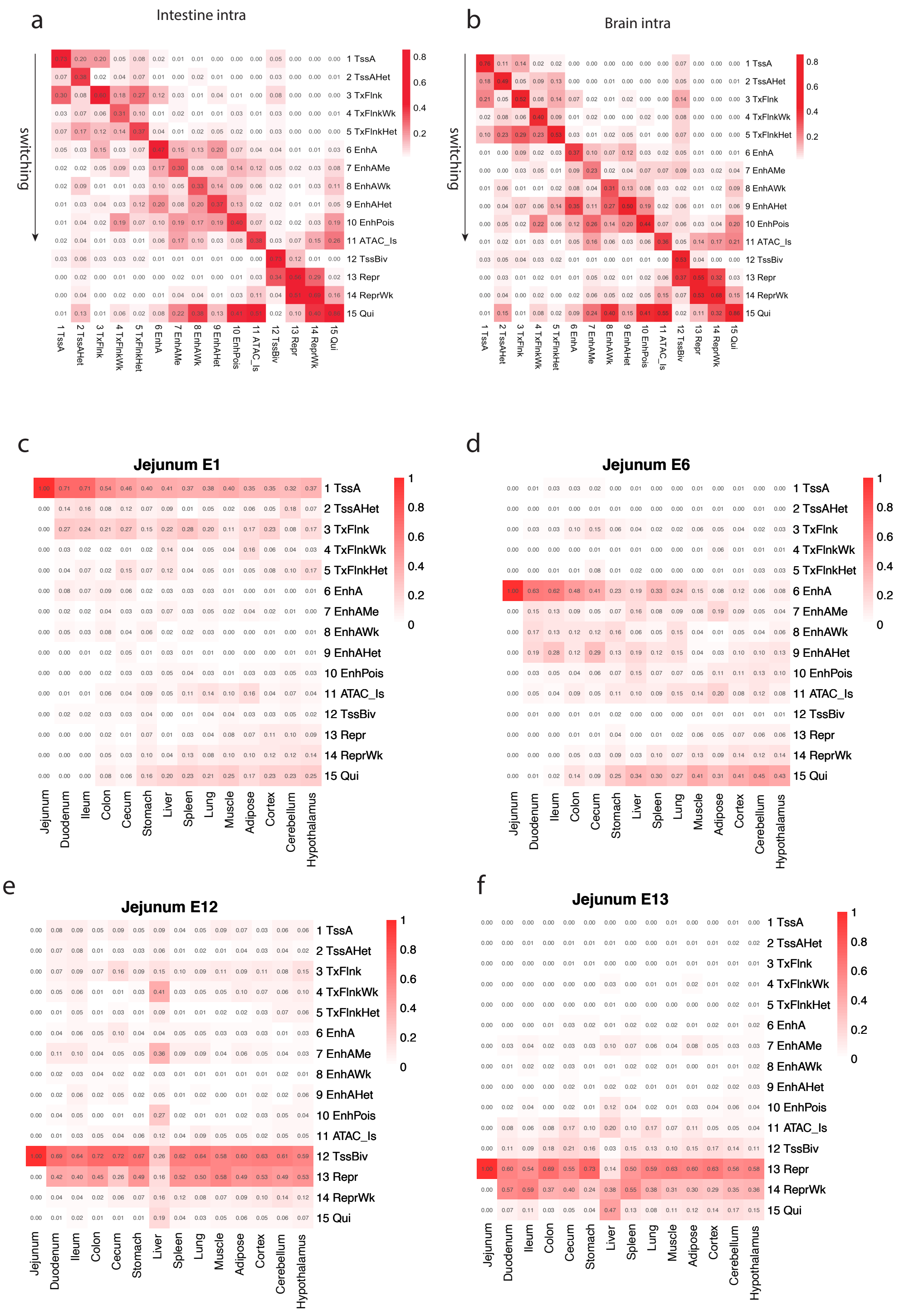

Supplementary Fig. 9 Chromatin states switching in tissues and TSE. **a,b** Chromatin states switching probabilities in between intestinal tissues, between brain tissues **c,d,e,f.** Chromatin

state switching for proximal-promoters (E1), enhancers(E6), TssBivs (E12), repressors (E13) of tissue-specifically expressed genes (TSE) in jejunum. E1: 5,000bp around TSE gene's TSS, E6, E12, and E13: 25,000bp according to chromatin state density peak in Fig. 2g.

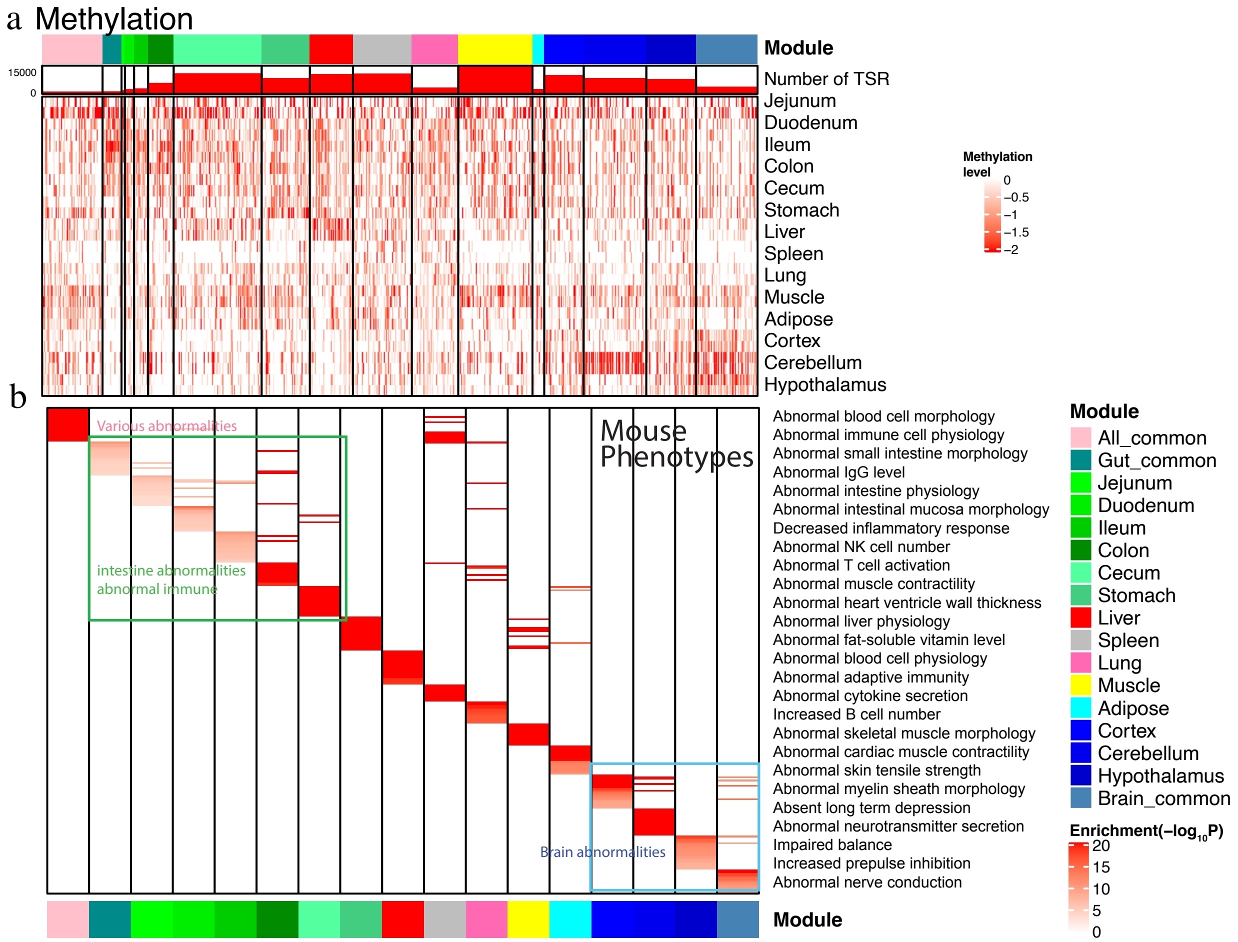

Supplementary Fig. 10 Methylation level and mouse phenotype enrichment of tissue-specific EnhAs. **a,** Methylation level of tissue-specific strong enhancers (EnhAs). The columns represent the genes in each module, the rows represent tissues. **b** Mouse phenotype enrichment of tissue-specific EnhAs. The columns represent 17 modules of strong enhancers. The rows represent phenotypes in each module. The *P* value was generated by GREAT[3] program.

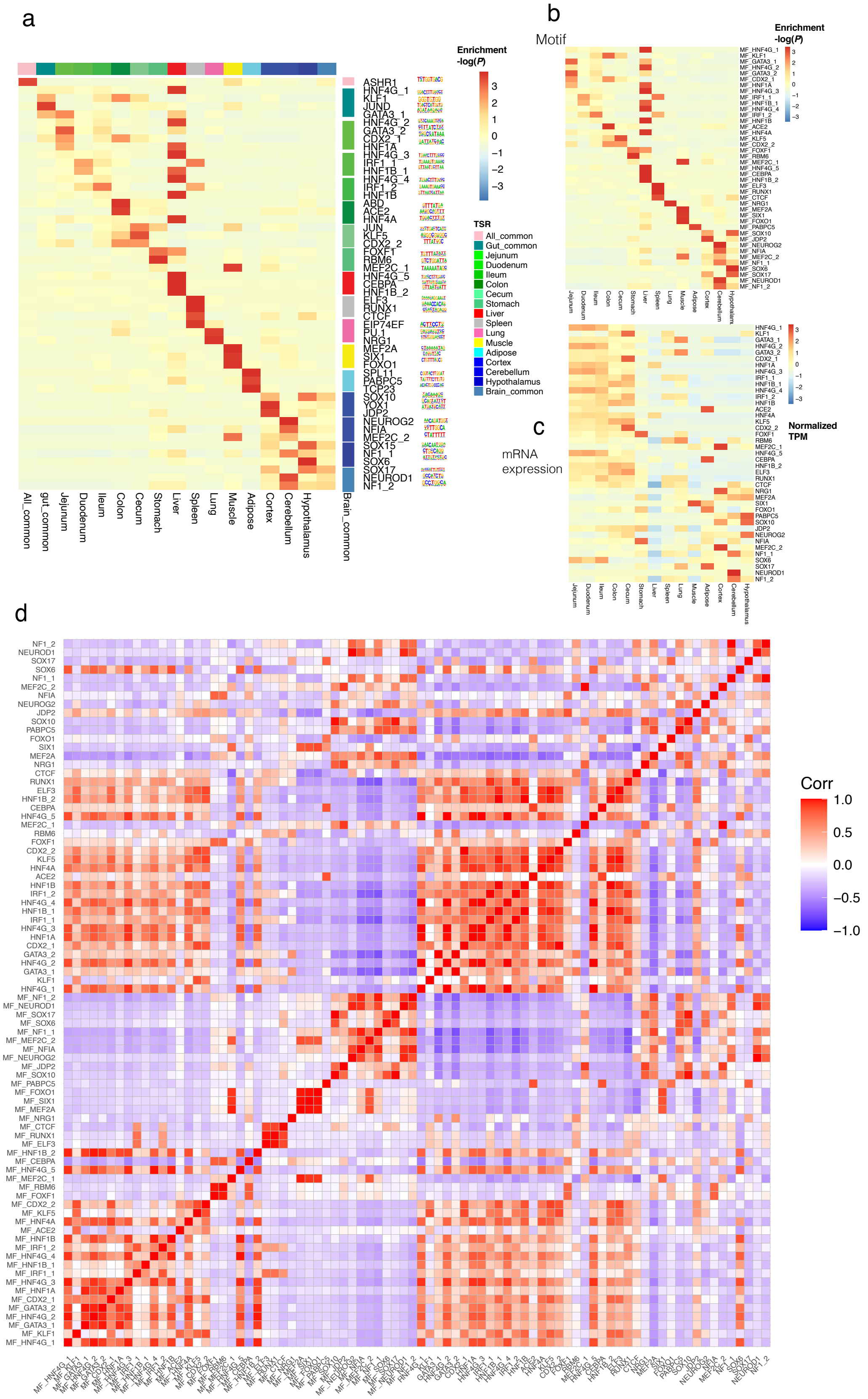
a
Enrichment -log(P)
3
2
1
0
−1
−2
−3
ASHR1
HNF4G_1
KLF1
JUND
GATA3_1
HNF4G_2
GATA3_2
CDX2_1
HNF1A
HNF4G_3
IRF1_1
HNF1B_1
HNF4G_4
IRF1_2
HNF1B
ABD
ACE2
HNF4A
JUN
KLF5
CDX2_2
FOXF1
RBM6
MEF2C_1
HNF4G_5
CEBPA
HNF1B_2
ELF3
RUNX1
CTCF
EIP74EF
PU.1
NRG1
MEF2A
SIX1
FOXO1
SPL11
PABPC5
TCP23
SOX10
YOX1
JDP2
NEUROG2
NFIA
MEF2C_2
SOX15
NF1_1
SOX6
SOX17
NEUROD1
NF1_2
All_common
gut_common
Jejunum
Duodenum
Ileum
Colon
Cecum
Stomach
Liver
Spleen
Lung
Muscle
Adipose
Cortex
Cerebellum
Hypothalamus
Brain_common
TSR
All_common
Gut_common
Jejunum
Duodenum
Ileum
Colon
Cecum
Stomach
Liver
Spleen
Lung
Muscle
Adipose
Cortex
Cerebellum
Hypothalamus
Brain_common
b
Motif
Enrichment -log(P)
c
mRNA expression
Normalized TPM
d
Corr
1.0
0.5
0.0
−0.5
−1.0

Supplementary Fig. 11 Motif enrichment of tissue-specific strong enhancers (EnhAs). **a,** Major motif enrichment in 14 tissues. The *P* value was generated by HOMMER. **b,c,** The corresponded motif enrichment and mRNA expression in each tissues. (Normalized and centered TPM). The *P* value was generated by HOMMER[4]. **d,** The correlation between motif enrichment and the mRNA expression of corresponding transcription factors' motif.

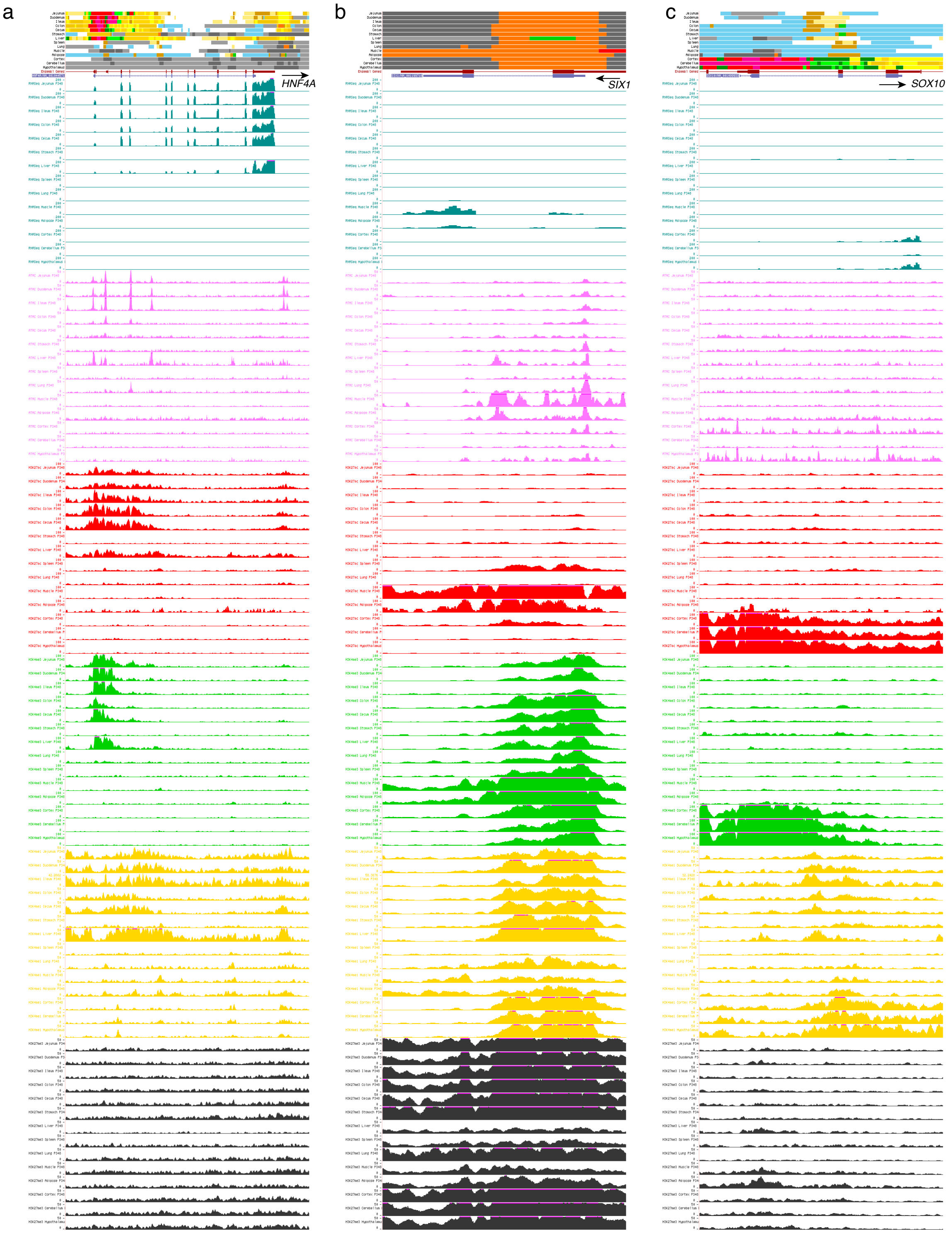

Supplementary Fig. 12 Transcription factors (TF) identified from tissue-specific EnhAs motifs.

a, Chromatin state around *HNF4A* gene (chr17:46,816,508-46,852,176) in 14 tissues. **b**,

Chromatin state around *SIX1* (chr1:189,619,374-189,625,708) in 14 tissues. c, Chromatin state around *SOX10* (chr5:9,889,423-9,903,021) in 14 tissues. Vertical scale 0-200 for RNA-seq, 0-100 for H3K27ac and H3K4me3, and 0-50 for other marks and ATAC-seq.

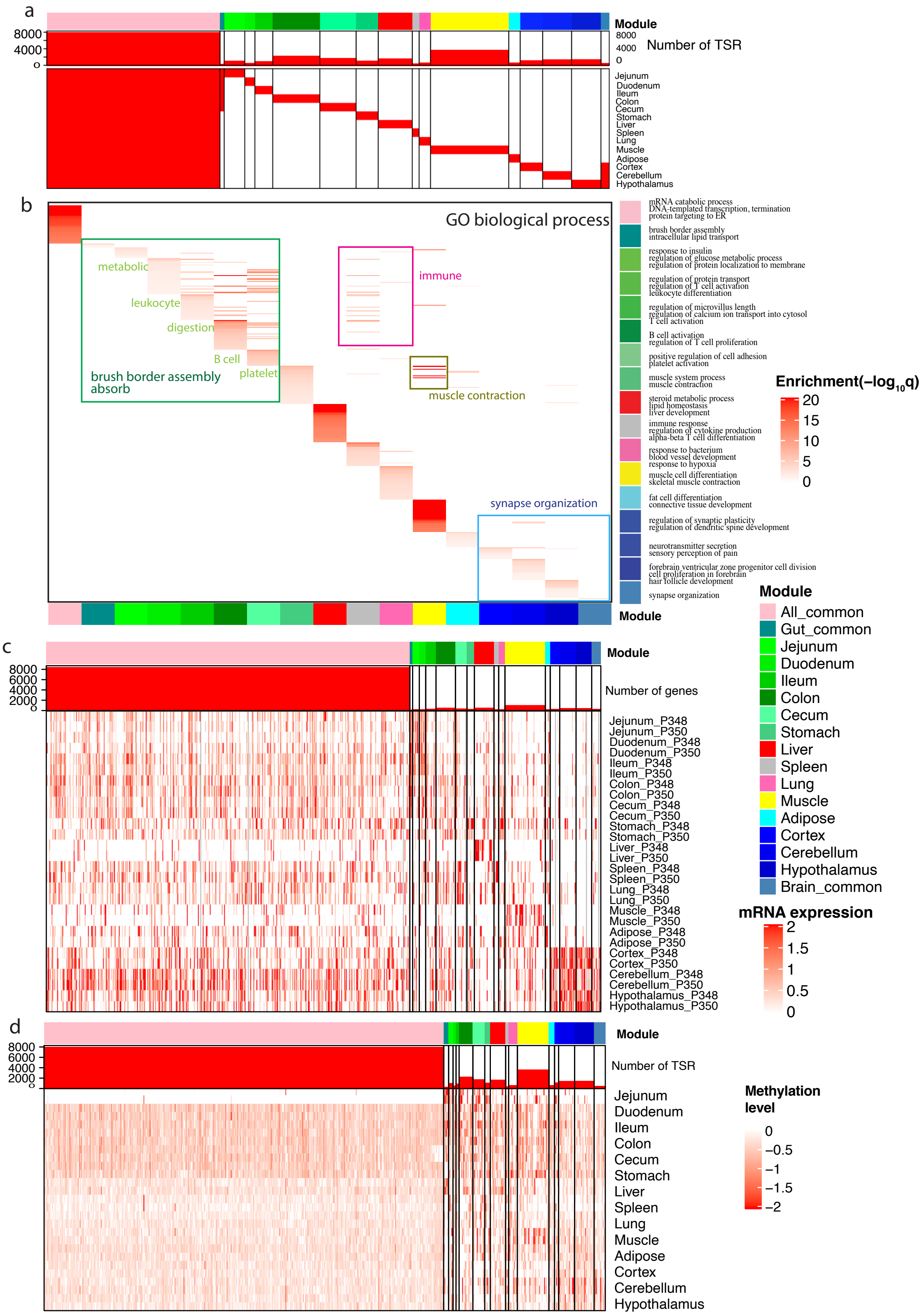

a
Module
Number of TSR
8000
4000
0
Jejunum
Duodenum
Ileum
Colon
Cecum
Stomach
Liver
Spleen
Lung
Muscle
Adipose
Cortex
Cerebellum
Hypothalamus
b
GO biological process
metabolic
leukocyte
digestion
B cell
platelet
brush border assembly
absorb
immune
muscle contraction
synapse organization
mRNA catabolic process
DNA-templated transcription, termination
protein targeting to ER
brush border assembly
intracellular lipid transport
response to insulin
regulation of glucose metabolic process
regulation of protein localization to membrane
regulation of protein transport
regulation of T cell activation
leukocyte differentiation
regulation of microvillus length
regulation of calcium ion transport into cytosol
T cell activation
B cell activation
regulation of T cell proliferation
positive regulation of cell adhesion
platelet activation
muscle system process
muscle contraction
steroid metabolic process
lipid homeostasis
liver development
immune response
regulation of cytokine production
alpha-beta T cell differentiation
response to bacterium
blood vessel development
response to hypoxia
muscle cell differentiation
skeletal muscle contraction
fat cell differentiation
connective tissue development
regulation of synaptic plasticity
regulation of dendritic spine development
neurotransmitter secretion
sensory perception of pain
forebrain ventricular zone progenitor cell division
cell proliferation in forebrain
hair follicle development
synapse organization
Enrichment(−log10q)
20
15
10
5
0
Module
Module
All_common
Gut_common
Jejunum
Duodenum
Ileum
Colon
Cecum
Stomach
Liver
Spleen
Lung
Muscle
Adipose
Cortex
Cerebellum
Hypothalamus
Brain_common
c
Module
Number of genes
8000
6000
4000
2000
0
Jejunum_P348
Jejunum_P350
Duodenum_P348
Duodenum_P350
Ileum_P348
Ileum_P350
Colon_P348
Colon_P350
Cecum_P348
Cecum_P350
Stomach_P348
Stomach_P350
Liver_P348
Liver_P350
Spleen_P348
Spleen_P350
Lung_P348
Lung_P350
Muscle_P348
Muscle_P350
Adipose_P348
Adipose_P350
Cortex_P348
Cortex_P350
Cerebellum_P348
Cerebellum_P350
Hypothalamus_P348
Hypothalamus_P350
mRNA expression
2
1.5
1
0.5
0
d
Module
Number of TSR
8000
6000
4000
2000
0
Jejunum
Duodenum
Ileum
Colon
Cecum
Stomach
Liver
Spleen
Lung
Muscle
Adipose
Cortex
Cerebellum
Hypothalamus
Methylation level
0
−0.5
−1
−1.5
−2

Supplementary Fig. 13 **Tissue-specific promoters (TssAs) and their potential functions in 14 tissues. a,** The number and enrichment distribution of 17 modules of promoters in tissues. TSR: tissue-specific regulatory elements. **b**, Functional enrichment of proximal (2000bp) genes for each module based on gene ontology (GO) biological processes. **c,** The mRNA expression (TPM) of promoters' target genes (2000bp around transcription start sites) in each module. **d,** Methylation level of tissue-specific promoters.

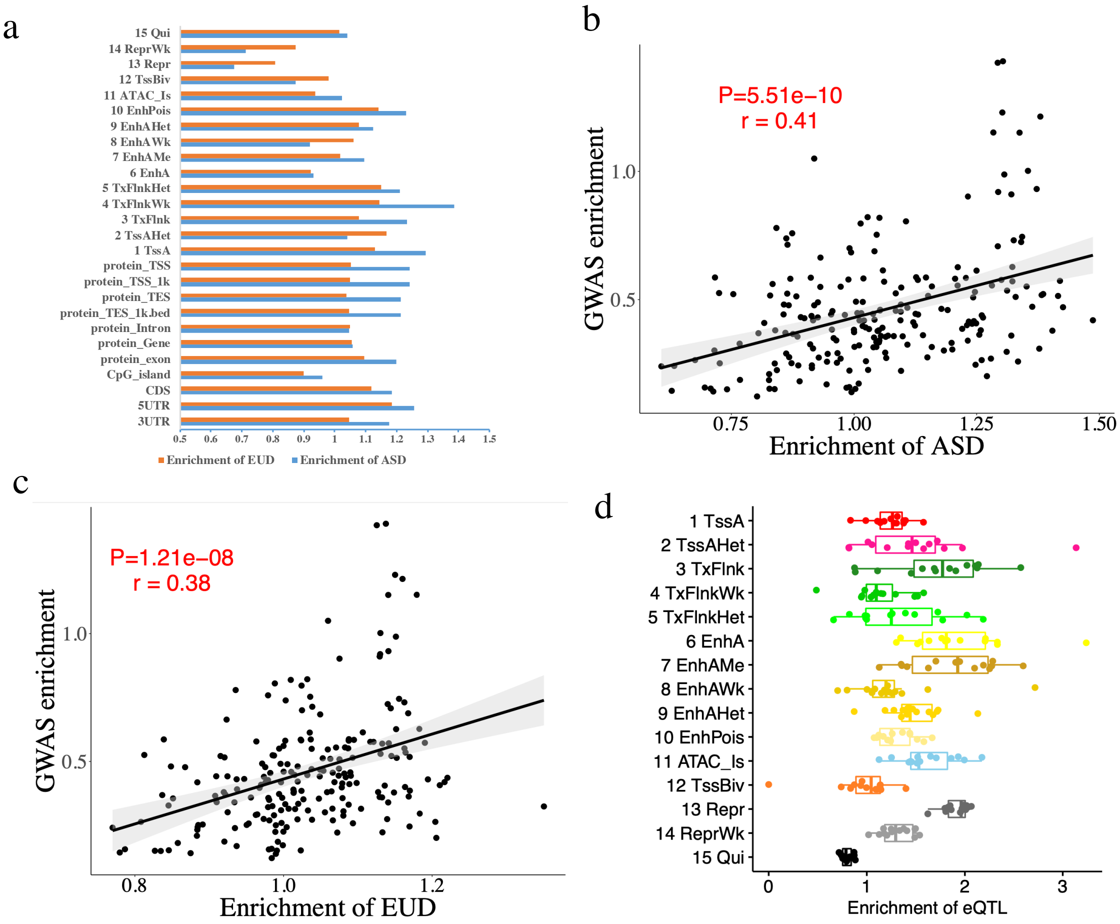

Supplementary Fig. 14 **Chromatin state enrichments of domestication selection signature and QTLs affecting complex traits in pigs. a,** Enrichment of selection signal of chromatin states compared with gene elements. ASD: Asian pig domestication; EUD: European pig domestication. **b,c,** Correlations among GWAS enrichment (44 traits) and domestication selection signature enrichment of chromatin states in tissues. The significant test was bases on the two-sided *t*-test. **d**, Porcine muscle eQTL enrichment of chromatin states in 14 tissues. Whiskers show 1.5× interquartile range. Each circle represents one of 14 different tissues.

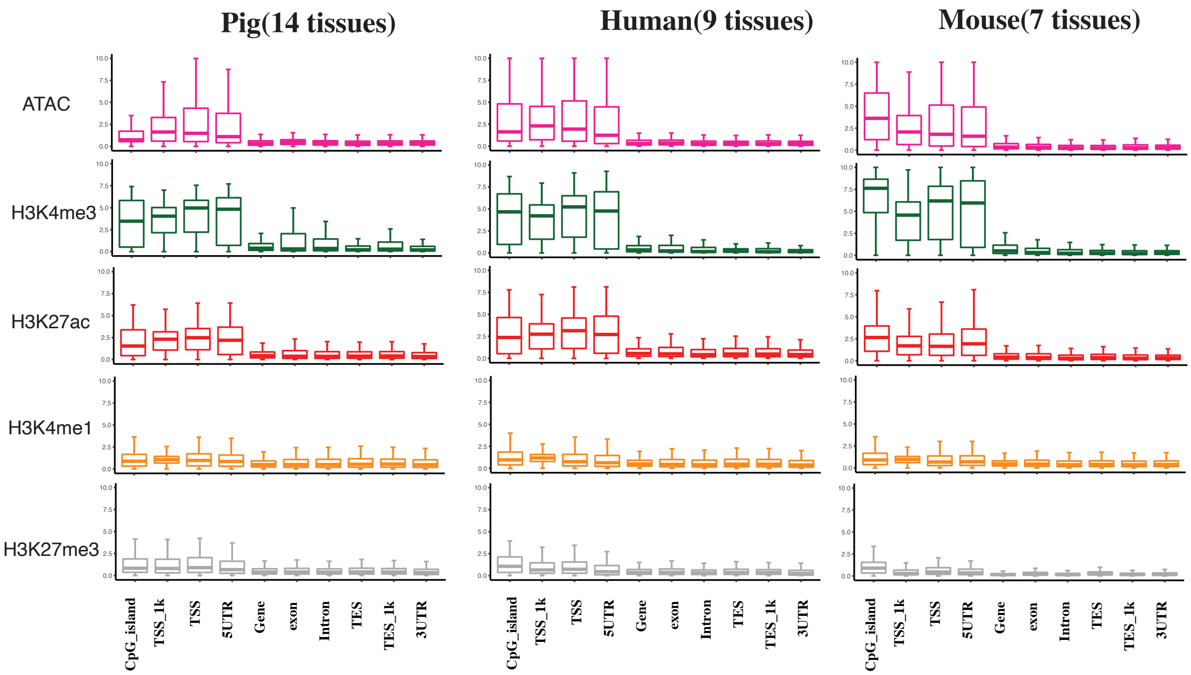

Supplementary Fig. 15 Traces of evolutionary conservation of epigenomes. Mark signal of gene elements among pig (14 tissues), human (9 tissues), and mouse (7 tissues). Error bars represent standard error of mark signal among tissues. Whiskers show 1.5× interquartile range.

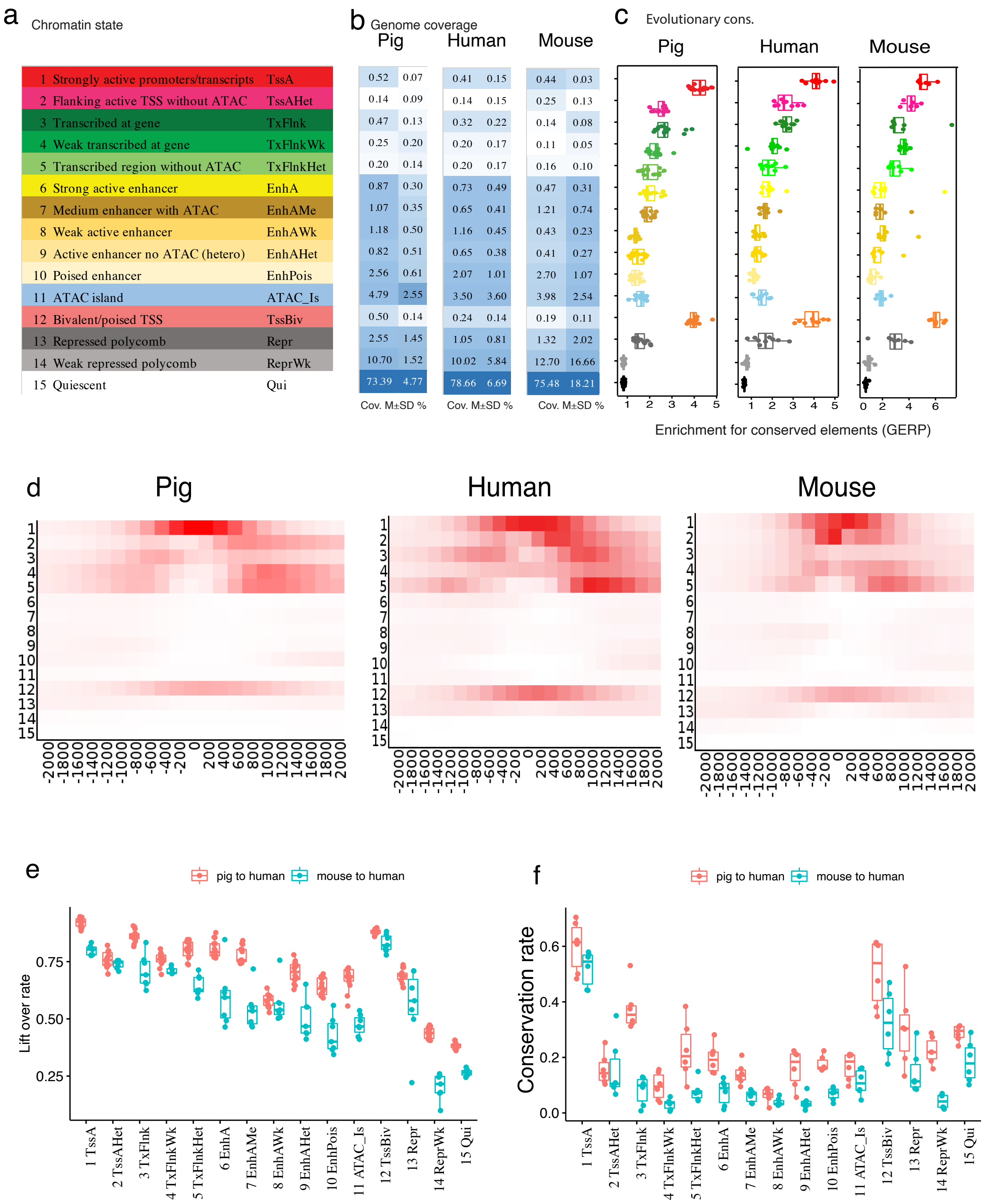
a
Chromatin state
1 Strongly active promoters/transcripts TssA
2 Flanking active TSS without ATAC TssAHet
3 Transcribed at gene TxFlnk
4 Weak transcribed at gene TxFlnkWk
5 Transcribed region without ATAC TxFlnkHet
6 Strong active enhancer EnhA
7 Medium enhancer with ATAC EnhAMe
8 Weak active enhancer EnhAWk
9 Active enhancer no ATAC (hetero) EnhAHet
10 Poised enhancer EnhPois
11 ATAC island ATAC_Is
12 Bivalent/poised TSS TssBiv
13 Repressed polycomb Repr
14 Weak repressed polycomb ReprWk
15 Quiescent Qui
b
Genome coverage
Pig
Human
Mouse
0.52 0.07 0.41 0.15 0.44 0.03
0.14 0.09 0.14 0.15 0.25 0.13
0.47 0.13 0.32 0.22 0.14 0.08
0.25 0.20 0.20 0.17 0.11 0.05
0.20 0.14 0.20 0.17 0.16 0.10
0.87 0.30 0.73 0.49 0.47 0.31
1.07 0.35 0.65 0.41 1.21 0.74
1.18 0.50 1.16 0.45 0.43 0.23
0.82 0.51 0.65 0.38 0.41 0.27
2.56 0.61 2.07 1.01 2.70 1.07
4.79 2.55 3.50 3.60 3.98 2.54
0.50 0.14 0.24 0.14 0.19 0.11
2.55 1.45 1.05 0.81 1.32 2.02
10.70 1.52 10.02 5.84 12.70 16.66
73.39 4.77 78.66 6.69 75.48 18.21
Cov. M±SD %
Cov. M±SD %
Cov. M±SD %
c
Evolutionary cons.
Pig
Human
Mouse
Enrichment for conserved elements (GERP)
d
Pig
Human
Mouse
e
pig to human
mouse to human
Lift over rate
1 TssA
2 TssAHet
3 TxFlnk
4 TxFlnkWk
5 TxFlnkHet
6 EnhA
7 EnhAMe
8 EnhAWk
9 EnhAHet
10 EnhPois
11 ATAC_Is
12 TssBiv
13 Repr
14 ReprWk
15 Qui
f
pig to human
mouse to human
Conservation rate

122

Supplementary Fig. 16 Chromatin state conservation in pig, human, and mouse. **a,** 15 chromatin states in three species genomes defined by the ChromHMM. **b,** Genome coverage for each chromatin state in three species. **c,** Fold enrichments of chromatin states for mammalian conserved elements (GERP) in three species. Whiskers show 1.5× interquartile range. Circles are 14 different tissues. **d,** Enrichment of chromatin state around genes in three species. **e,** The difference of lift over rate for each chromatin state between pig to human and mouse to human. Whiskers show 1.5× interquartile range. Circles are 14 different tissues. **f,** The difference of conservation rate of each chromatin state between pig to human and mouse to human. Whiskers show 1.5× interquartile range. Each circle represents one of 14 different tissues.

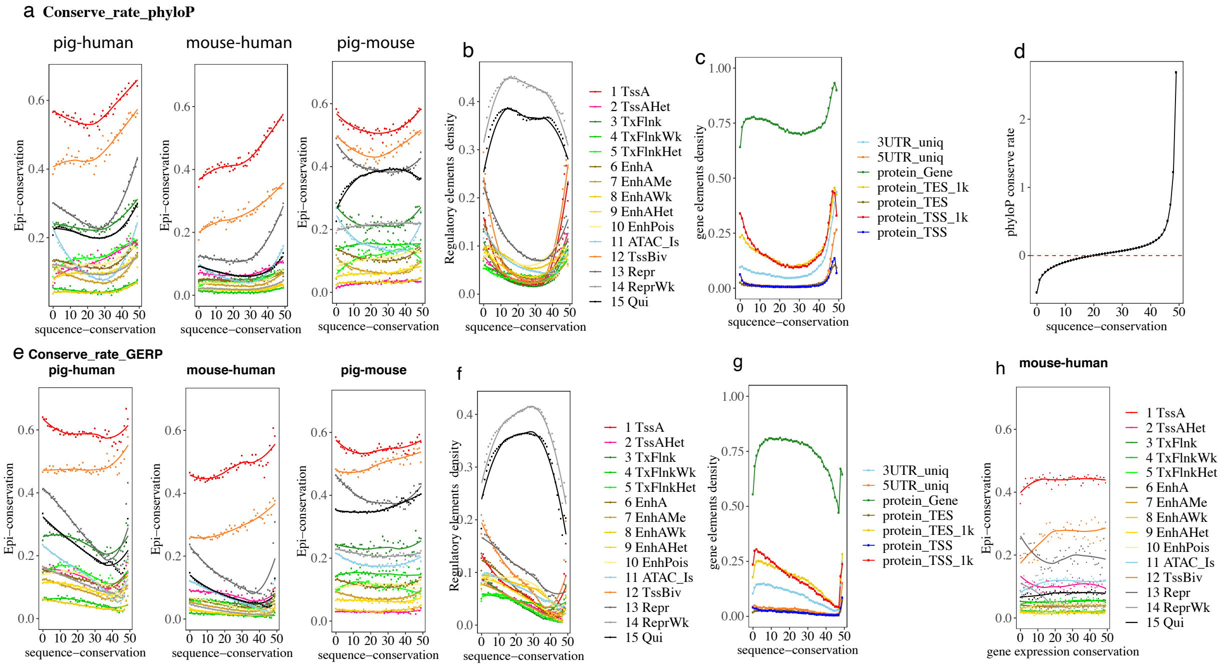

Supplementary Fig.17 Global comparison of genomic and epigenomic conservations. **a,** The relations between sequence conservation (phyloP conservation rate) and epigenomic

conservation across 6 tissues. These segments were ordered from the fastest changing (0th), to slowest changing (49th). **b,** Regulatory elements density in different sequence conservation segments. **c,** Gene elements densities in different sequence conservation segments. **d**, Average phyloP conservation rate in each segment. **e,** The relations between sequence conservation (GERP conservation rate) and epigenomic conservation. **f,g,** Regulatory and gene elements in different GERP conservation segments. **h**, Relationship between expression conservation and epigenomic conservation across 6 tissues in mouse-human pair. Expression conservation was based on expression of 14,302 orthologous genes among 3 species. Regions were ordered from the greatest difference (0th), to smallest difference (49th) in expression.

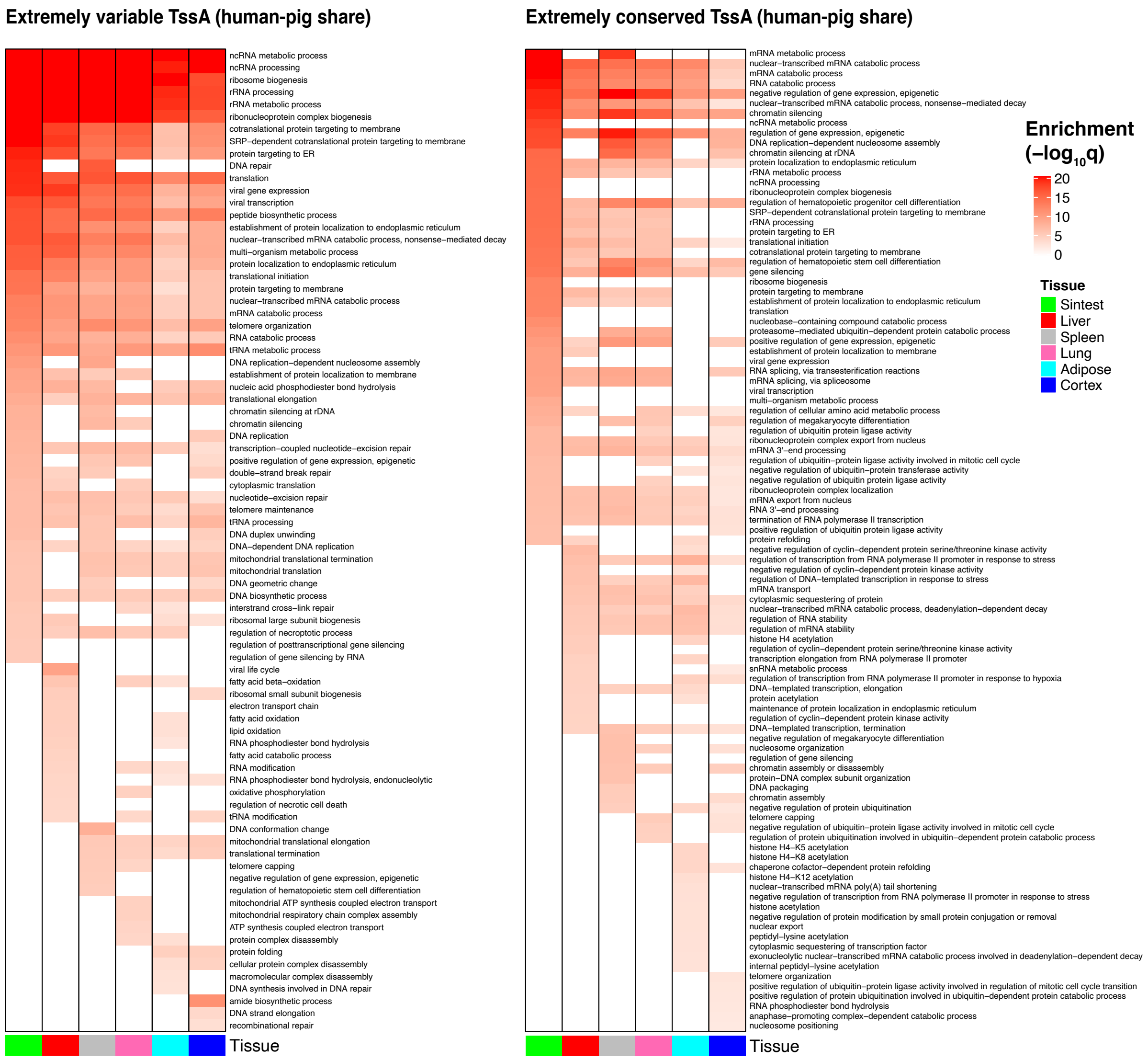

Supplementary Fig. 18 GO functional enrichments of TssA regulatory elements for sequences extremely variable (0th) and extremely conserved regions (49th).

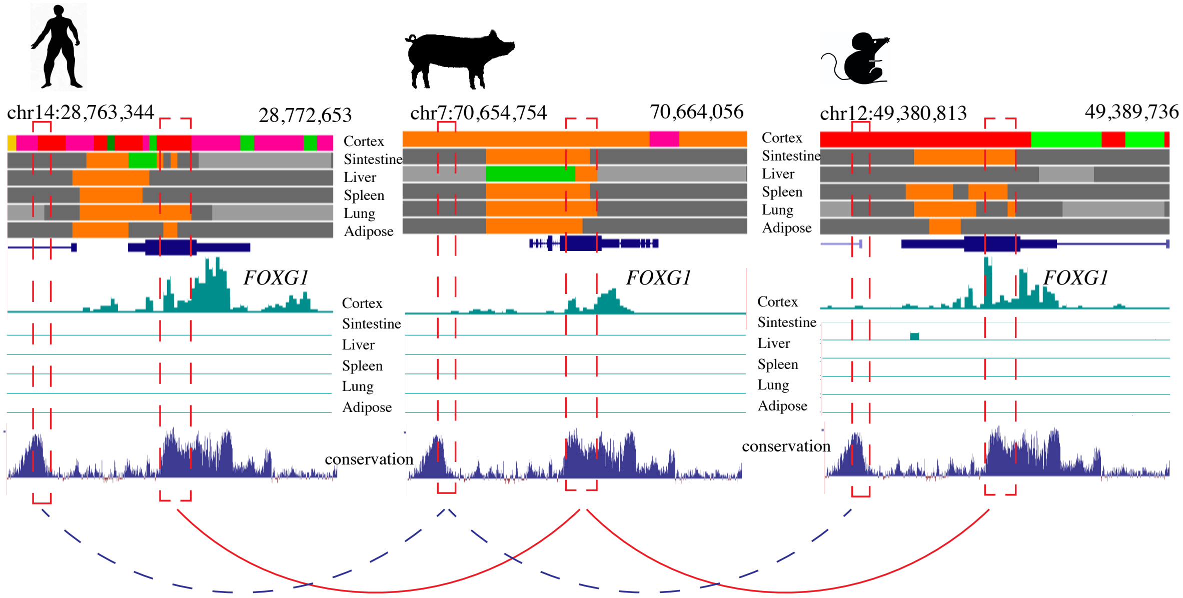

Supplementary Fig. 19 Chromatin state, mRNA expression, sequence conservation around *FOXG1* locus in pig, human and mouse. In sequence extremely conserved region, human *FOXG1* has two brain cortex-specific TssAs (red dashed rectangle boxes) in which they are TssABiv in pigs. Aim Forkhead Box G1(*FOXG1),* is very important gene in cortical cell fate related with autism spectrum disorders and other brain disease[5,6], showed higher *FOXG1*expression in human than that in pig brain cortex. Vertical scale 0-110 for RNA-seq.

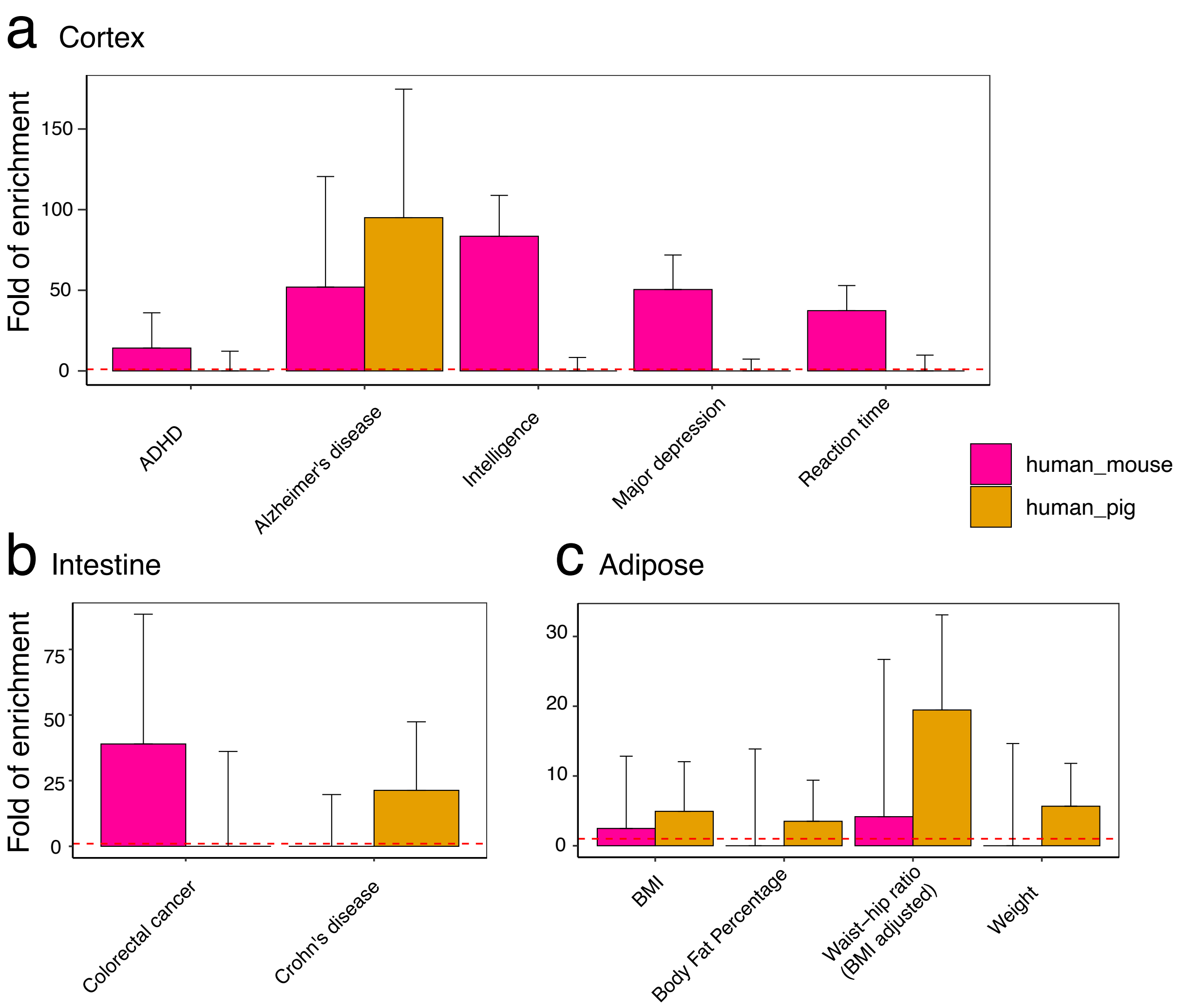

Supplementary Fig. 20 Different QTLs from human genome-wide association studies (GWAS) (47 human diseases and complex traits) enrichment between human-pig and human-mouse shared promoters (TssA) in brain cortex (2,439 vs. 1,614 TssA) (**a**), small intestine (2,975 vs. 1,779 TssA) (**b**), and adipose (2,583 vs. 1,046 TssA) (**c**) tissues. Dash line=1, above the dash line means significantly high enrichment. Error bars represent standard error around the estimates of enrichment. The data are available at https://doi.org/10.6084/m9.figshare.16531197.v1

1. Colin Kern, Y.W., Xiaoqin Xu, Zhangyuan Pan, Michelle Halstead, Kelly Chanthavixay, Perot Saelao, Susan Waters, Ruidong Xiang, Amanda Chamberlain, Ian Korf, Mary E. Delany, Hans H. Cheng, Juan F. Medrano, Alison L. Van Eenennaam, Chris K. Tuggle, Catherine Ernst, Paul Flicek, Gerald Quon, Pablo Ross, Huaijun Zhou. Functional genome annotations of three domestic animal species provide a vital resource for comparative and agricultural research. *Nature Communications* **12**, 1-11 (2021).
2. Foissac, S. *et al.* Multi-species annotation of transcriptome and chromatin structure in domesticated animals. *BMC Biology* **17**, 1-25 (2019).
3. McLean, C.Y. *et al.* GREAT improves functional interpretation of cis-regulatory regions. *Nat Biotechnol* **28**, 495-501 (2010).
4. Heinz, S. *et al.* Simple combinations of lineage-determining transcription factors prime cis-regulatory elements required for macrophage and B cell identities. *Molecular cell* **38**, 576-589 (2010).
5. Mariani, J. *et al.* FOXG1-dependent dysregulation of GABA/glutamate neuron differentiation in autism spectrum disorders. *Cell* **162**, 375-390 (2015).
6. Hanashima, C., Li, S.C., Shen, L., Lai, E. & Fishell, G. Foxg1 suppresses early cortical cell fate. *Science* **303**, 56-59 (2004).
