# Supplementary material for: Pig genome functional annotation enhances the biological interpretation of complex traits and human disease
Source: Nat Commun. 2021 Oct 6;12:5848. doi: 10.1038/s41467-021-26153-7 (PMC8494738; doi:10.1038/s41467-021-26153-7)
Supplement: Supplementary file 3 — Description of Additional Supplementary Files [file 41467_2021_26153_MOESM3_ESM.pdf]

Description of Additional Supplementary Files

Supplementary Data 1. The summary of data quality of all the ChIP-seq of epi-marks, control, ATAC-seq, RRBS and RNA-seq data sets of pig.

Supplementary Data 2. Chromatin state number and genome coverage in 14 tissues.

Supplementary Data 3. Gene Ontology (GO) enrichment of genes in ChrX of module 1 of large-scale chromatin.

Supplementary Data 4. Gene Ontology (Go) enrichment of tissue specific expression genes.

Supplementary Data 5. A summary of target gene prediction of strong enhancer (EnhA).

Supplementary Data 6. Gene Ontology (Go) of putative targeted genes of tissue-specific EnhAs.

Supplementary Data 7. Human phenotype enrichment of putative targeted genes of tissue-specific EnhAs.

Supplementary Data 8. Gene Ontology (GO) of putative targeted genes of tissue-specific TssAs.

Supplementary Data 9. Whole genome sequencing data using to detect selection signatures.

Supplementary Data 10. Top 5% of selection signatures in Asian local pig compare with wild pig.

Supplementary Data 11. Top 5% of selection signatures in European local pig compare with wild pig.

Supplementary Data 12. The data summary of pig GWAS used in this study.

Supplementary Data 13. The summary of data quality of ChIP-seq, control, ATAC-seq, RRBS and RNA-seq data sets in human.

Supplementary Data 14. The summary of data quality of ChIP-seq, control, ATAC-seq, RRBS and RNA-seq data sets in mouse.

Supplementary Data 15. Gene Ontology (GO) enrichment of putative targeted genes of human-specific TssAs.

Supplementary Data 16. Human GWAS data used in this study.

Supplementary Data 17. LDSC regression results of base model.
